# Hippocampal Adaptations to Continuous Aerobic Training: A Functional and Ultrastructural Evaluation in a Young Murine Model

**DOI:** 10.3390/jfmk6040101

**Published:** 2021-12-08

**Authors:** Ida Cariati, Roberto Bonanni, Gabriele Pallone, Manuel Scimeca, Claudio Frank, Virginia Tancredi, Giovanna D’Arcangelo

**Affiliations:** 1 Department of Clinical Sciences and Translational Medicine, “Tor Vergata” University of Rome, Via Montpellier 1, 00133 Rome, Italy; roberto.bonanni1288@gmail.com; 2 Department of System Medicine, “Tor Vergata” University of Rome, Via Montpellier 1, 00133 Rome, Italy; gabriele.pallone@gmail.com (G.P.); tancredi@uniroma2.it (V.T.); giovanna.darcangelo@uniroma2.it (G.D.); 3 Department of Biomedicine and Prevention, “Tor Vergata” University of Rome, Via Montpellier 1, 00133 Rome, Italy; manuel.scimeca@uniroma2.it; 4 UniCamillus-Saint Camillus International University of Health Sciences, Via di Sant’Alessandro 8, 00131 Rome, Italy; claudio.frank@unicamillus.org; 5 Centre of Space Bio-Medicine, “Tor Vergata” University of Rome, Via Montpellier 1, 00133 Rome, Italy

**Keywords:** aerobic exercise, synaptic plasticity, hippocampus, training protocols, cognitive decline

## Abstract

Aerobic training is known to influence cognitive processes, such as memory and learning, both in animal models and in humans. Particularly, in vitro and in vivo studies have shown that aerobic exercise can increase neurogenesis in the dentate gyrus, improve hippocampal long-term potentiation (LTP), and reduce age-related decline in mnemonic function. However, the underlying mechanisms are not yet fully understood. Based on this evidence, the aim of our study was to verify whether the application of two aerobic training protocols, different in terms of speed and speed variation, could modulate synaptic plasticity in a young murine model. Therefore, we assessed the presence of any functional changes by extracellular recordings in vitro in mouse hippocampal slices and structural alterations by transmission electron microscopy (TEM). Our results showed that an aerobic training protocol, well designed in terms of speed and speed variation, significantly contributes to improving synaptic plasticity and hippocampal ultrastructure, optimizing its benefits in the brain. Future studies will aim to clarify the underlying biological mechanisms involved in the modulation of synaptic plasticity induced by aerobic training.

## 1. Introduction

Physical exercise has positive effects on general health and reduces the incidence of pathological conditions such as diabetes, osteoporosis, cardiovascular diseases, obesity, and other chronic disorders [1,2,3]. The positive effects of exercise on brain activity have long been discussed, although only recently scientific evidence based on neuroimaging approaches demonstrated the effectiveness of physical activity in improving cognitive health across the human lifespan [4,5].

The beneficial effects of exercise, particularly aerobic exercise, on the brain and behavior were initially studied in animal models and focused largely on the impact of exercise on hippocampal structure, which plays a key role in learning and memory formation [6,7,8]. Evidence has suggested that wheel running and treadmill training improve spatial learning in rodents and promote increased neuron density in the hippocampal areas CA1 and CA3 [9,10,11]. Furthermore, aerobic exercise is known to increase cell proliferation and neurogenesis in the dentate gyrus, as well as improve synaptic plasticity and spatial learning in both rats and mice [12,13,14]. Interestingly, exercise-induced changes in the hippocampus were associated with improved performance in spatial memory tasks [15].

Similar results have been found in human studies, showing that aerobic exercise increases hippocampal volume and reduces age-related decline in memory function [16,17,18]. In addition, several intervention studies have exhibited improved cognitive performance in elderly subjects undergoing a physical activity program that produces significant increases in cardiorespiratory fitness, strongly supporting the impact of training on cognitive processes [19].

Over the decades, our knowledge of the neuronal and molecular processes of memory has greatly improved, providing a basis for the identification of therapeutic strategies to slow and/or prevent age-related cognitive decline in humans [20,21]. Among these, exercise has been suggested as an effective non-pharmacological approach to preserve cognitive function and treat neurodegenerative and/or psychiatric conditions [22]. In this regard, Do et al. recently studied the effects of voluntary exercise on hypothalamic neurodegeneration in a mouse model of Alzheimer’s disease, in which metabolic abnormalities, such as increased energy expenditure through enhanced oxygen consumption and increased caloric intake, were observed prior to the accumulation of amyloid plaques [23]. Interestingly, the authors observed a significant reduction in the expression of inflammatory and apoptotic markers in the hypothalamus of mice subjected to 4 weeks of voluntary wheeled exercise, suggesting a hypothalamus-mediated mechanism whereby exercise could counteract Alzheimer’s disease-related neurodegeneration [23].

In recent years, several mechanisms have been proposed to explain the positive impacts of aerobic exercise, including increased cerebral blood flow, changes in neurotransmitter release, structural changes in the central nervous system (CNS), and altered arousal levels [24]. A more recent proposal points to neurotrophic factors as possible agonists in facilitating improved motor performance [25]. Among these, brain-derived neurotrophic factor (BDNF) could play a key role, as observed in previous studies showing that motor performance in rat models with middle cerebral artery occlusion was impaired following pharmacological interruption of BDNF production or, conversely, improved when BDNF production was enhanced [26,27].

Importantly, regular physical activity is now generally accepted to promote the release of myokines and metabolites into the circulation, which can cross the blood–brain barrier at the level of brain capillaries and influence the functions of neurons and glial cells, thus modifying neurotransmission in different regions of the brain [28]. In this regard, an important role has recently been attributed to irisin, a myokine produced by cleavage of the precursor fibronectin type III domain-containing 5 (FNDC5) during exercise [29]. Particularly, Lourenco et al. observed a reduced expression of FNDC5/irisin in the hippocampus and cerebrospinal fluid of animal models of Alzheimer’s disease, correlated with a significant impairment of long-term potentiation (LTP), a phenomenon of synaptic plasticity, and object recognition memory [30]. Surprisingly, increased FNDC5/irisin levels promoted improved synaptic plasticity and counteracted memory impairment, highlighting the protective role of exercise in neurodegeneration [30].

Despite the latest evidence, prescribing specific exercises to maximize their positive effects on cognitive processes is not yet possible, because the levels of molecules released during muscle contraction change during and after exercise. In addition, it is not yet clear how brain functioning can vary with the type, intensity, and timing of exercise [22].

Based on this evidence, the aim of our work was to verify whether aerobic training can modulate synaptic plasticity in a young murine model, evaluating the presence of any functional changes by extracellular in vitro recordings in mouse hippocampal slices and structural alterations by transmission electron microscopy (TEM). Therefore, we applied two different continuous aerobic training protocols to assess whether any effects observed at the hippocampal level could depend on the use of different training protocols in terms of speed and speed variation.

## 2. Materials and Methods

### 2.1. Animals

Eighteen 1-month-old male mice, belonging to the wild-type BALB/c strain, were used, following the procedures established by the European Union Council Directive 2010/63/EU for animal experiments [31]. All experimental protocols were approved by the Italian Ministry of Public Health (authorization no. 86/2018-PR).

Animals were divided into two groups (five mice per group), each subjected to a different aerobic training protocol, and a third control group (eight mice), which did not perform any type of training. The health status of animals was monitored daily by resident veterinarians and experimenters, considering weight, coat and skin condition, and body functions. All experimental animals were kept under the same housing conditions and diet.

### 2.2. Training Protocols

The two experimental groups underwent aerobic training using a RotaRod (Cat N 47600, Ugo Basile srl, Milan, Italy). It features 5 cylinders with a diameter of 3 cm and a circumference of 9.42 cm covered by rubber to ensure an optimal grip for the rodents. A total of 6 panels with a diameter of 25 cm divided the 5 lanes, each with a 57 mm width, allowing 5 animals to run simultaneously. An attached display showed the types and speeds of rotation, the time elapsed since the start of the training session, and the time since the last fall. Finally, a control panel allowed the angular speed to be varied within a range (2–80 laps for minute, RPM) and the time intervals for the increasing speed modes from 6 sec to 10 min.

We administered two aerobic training protocols, progressive continuous (PC) and uniform continuous (UC), differing in terms of speed and speed variations, as described in our previous work [32] and summarized in Table 1. The PC protocol consisted of 18 min of training at a gradual speed of rotation, increasing from low to high intensity (10–32 RPM). During the UC training protocol, a rate of 13 RPM was set for 26 min. Training sessions were conducted three times a week for 12 weeks, for a total of 36 days of activity. The animals were raised on a light/dark cycle of 12/12 h, and training was carried out in the morning, between 10:00 and 11:00 a.m.

### 2.3. Extracellular Recordings in Mouse Hippocampal Slices

The animals belonging to the different experimental groups were sacrificed after 12 weeks of training, as were the sedentary animals. All efforts were made to minimize the number of animals used and their suffering. Under anesthesia with halothane (2-Brom-2-chlor-1,1,1-trifuor-ethan), mice were sacrificed, and their brains were quickly removed and placed in cold, oxygenated artificial cerebral spinal fluid (ACSF) containing the following (in mM): NaCl, 124; KCl, 2; KH_2_PO_4_, 1.25; MgSO_4_, 2; CaCl_2_, 2; NaHCO_3_, 26; and glucose, 10. The hippocampus was rapidly dissected and cut transversely into 450 μm thick slices using a McIlwain tissue chopper (Mickle Laboratory Engineering Co., Gomshall, UK). Then, hippocampal slices were transferred to a tissue chamber, where they were laid in an interface between oxygenated ACSF and humidified gas (95% O_2_, 5% CO_2_) at 32–34 °C (pH = 7.4), constantly superfused at flow rate of 1.2 mL/min.

Extracellular recordings of the population spikes (PSs) were made in the stratum pyramidale of the CA1 subfield, with glass microelectrodes filled with 2 M NaCl (resistance 5–10 MΩ). Orthodromic stimuli (10–500 mA, 20–90 ms, 0.1 Hz) were delivered through a platinum electrode placed in the stratum radiatum (Schaffer collaterals). The test stimulus intensity of 50 ms square pulses was adjusted to give a PS of 2–4 mV at 0.03 Hz. The PS amplitude was calculated every minute as the average of six recordings performed every 10 s. A high-frequency stimulation (HFS, 100 Hz, 1 s), after the recording of stable signals (15–20 min), was given to assess changes in PS amplitude, which was expressed as a percentage of the basal PS amplitude. Signals were fed to an Axoclamp-2A amplifier (Foster City, CA, USA), acquired through a digital/analogic system (Digidata 1440A, Axon Instruments, Foster City, CA, USA) and analyzed with pCLAMP10 software (Axon Instruments, Foster City, CA, USA).

### 2.4. TEM Evaluation

For TEM evaluation, 1 mm^3^ of hippocampal tissue from cerebral biopsies was fixed in 4% paraformaldehyde and post-fixed in 2% osmium tetroxide [33]. After washing with 0.1 M phosphate buffer, the sample was dehydrated by a series of incubations in 30%, 50%, and 70% ethanol. Dehydration was continued by incubation steps in 95% ethanol, absolute ethanol, and propylene oxide, after which samples were embedded in Epon (Agar Scientific Ltd., Parsonage Lane, Stansted, Essex CM24 8GF, UK) [34]. Ultra-thin sections, 80 nm thick, were mounted on copper grids and examined with a transmission electron microscope (Model JEM-1400 series 120 kV, JEOL USA, Inc. 11 Dearborn Road Peabody, MA, USA).

### 2.5. Statistical Analysis

Statistical analysis was performed using GraphPad Prism 8 Software (Prism 8.0.1, La Jolla, CA, USA). For electrophysiological experiments, data were expressed as the mean ± SEM, with *n* representing the number of slices analyzed. Data were compared with two-way ANOVA and Tukey’s multiple comparison tests and were considered significantly different if *p* < 0.05.

## 3. Results

### 3.1. Synaptic Plasticity Following Continuous Aerobic Training

The effects of two continuous aerobic training protocols, differing in terms of speed and speed variation, on the synaptic plasticity expression were analyzed in the CA1 region of hippocampal slices from trained mice compared to sedentary mice which did not perform any type of training.

**Figure 1:**
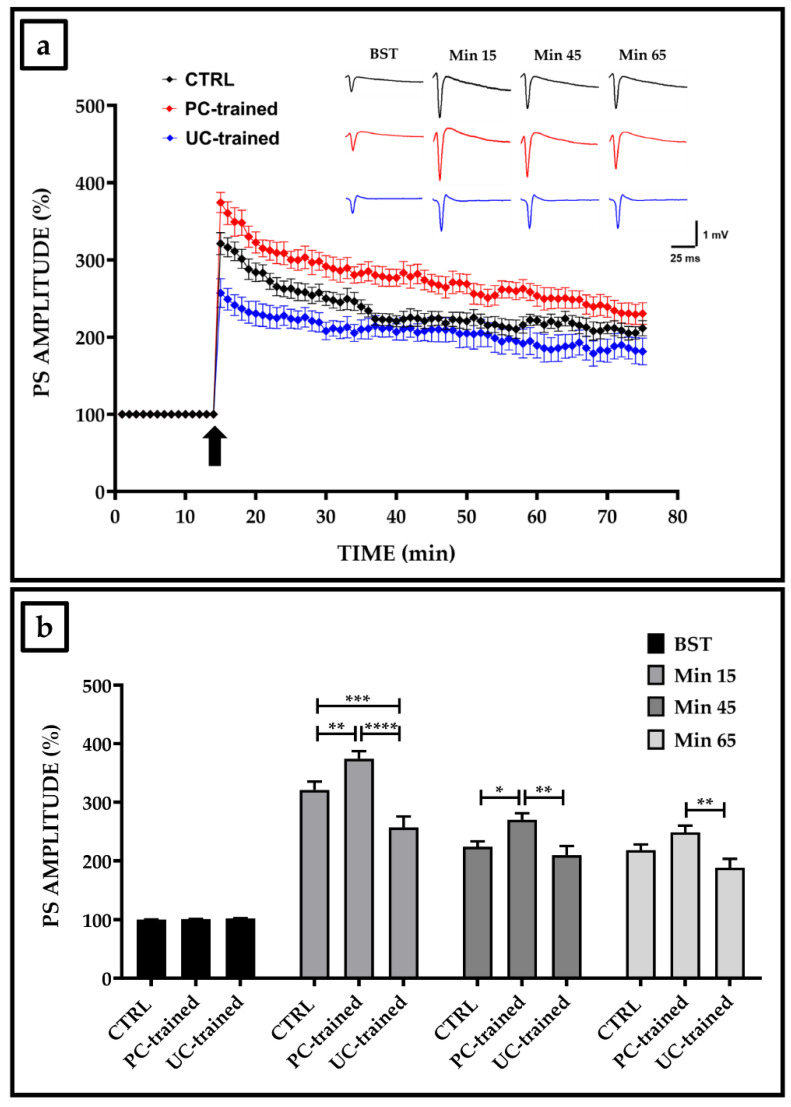
Synaptic plasticity in the CA1 hippocampal subfield of trained and sedentary mice. (**a**) Percentage population spike (PS) amplitude as a function of time after high-frequency stimulation (HFS), applied at time t = 15 (arrow), is shown in CTRL (black line, *n* = 15), in PC-trained (red line, *n* = 9), and in UC-trained (blue line, *n* = 8) mice slices. The insert shows representative recordings obtained from slices of each experimental group. The first curve of each group refers to the basal synaptic transmission (BST) and it was recorded before the HFS application, whereas the other curves refer to PS at times 15, 45 and 65 min after the HFS. (**b**) The PS amplitude values of BST, at min 15 (immediately after HFS), at min 45 and at min 65 from the HFS, are shown for each experimental group. Bars in the plot are means ± SEM of values obtained from different slices. Note that a significant statistical difference was reported between trained and control groups at min 15 (CTRL vs. PC-trained, ** *p* < 0.01; CTRL vs. UC-trained, *** *p* < 0.001; PC-trained vs. UC-trained, **** *p* < 0.0001), at min 45 (CTRL vs. PC-trained, * *p* < 0.05; PC-trained vs. UC-trained, ** *p* < 0.01) and at min 65 (PC-trained vs. UC-trained, ** *p* < 0.01).

Figure 1a shows how the influence of training on synaptic plasticity varied depending on the protocol administered. Particularly, we obtained optimal results for the PC training protocol, which seemed to positively modulate synaptic plasticity throughout the electrophysiological recording, with significantly higher PS amplitude values than those of the other experimental groups. In contrast, no improvement in synaptic plasticity was observed in the hippocampal slices of mice trained with the UC protocol, which was inhibited in the first twenty minutes after HFS, whereas PS amplitude values remained stable until the end of the electrophysiological recording with values similar to those of the CTRL group.

Figure 1b shows the following PS amplitude values at four different experimental times and their significance: basal synaptic transmission (BST), before HFS (CTRL: 100.7 ± 0.4, PC-trained: 101.0 ± 0.2, UC-trained: 102.1 ± 0.3); at min 15, immediately after HFS (CTRL: 321.0 ± 14.3, PC-trained: 374.2 ± 13.0, UC-trained: 257.0 ± 18.7; CTRL vs. PC-trained, ** *p* < 0.01; CTRL vs. UC-trained, *** *p* < 0.001; PC-trained vs. UC-trained, **** *p* < 0.0001); at min 45 (CTRL: 224.0 ± 9.4, PC-trained: 270.0 ± 13.0, UC-trained: 209.7 ± 15.7; CTRL vs. PC-trained, * *p* < 0.05; PC-trained vs. UC-trained, ** *p* < 0.01); and at min 65 (CTRL: 218.1 ± 10.0, PC-trained: 248.6 ± 11.5, UC-trained: 188.7 ± 14.7; PC-trained vs. UC-trained, ** *p* < 0.01).

### 3.2. Ultrastructural Hippocampal Evaluation of the Sedentary and Trained Mice

TEM evaluation was performed to assess the presence of any relevant differences in hippocampal slices taken from sedentary and trained mice.

**Figure 2:**
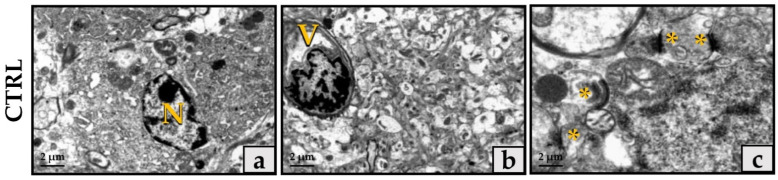
Ultrastructural evaluation by transmission electron microscopy (TEM) of the hippocampus of sedentary mice. (**a**) TEM evaluation of the hippocampus of CTRL mice not subjected to continuous aerobic training showed normal tissue organization with well-preserved nerve and glial cells. (**b**) Nerve processes were well represented, rich in neurotubules and neurofilaments, with slight vacuolization at the axonal level. (**c**) Synapses were well represented with well-preserved morphology. Scale bar represents 2 μm (N: nucleus; V: vessel; *: synapse).

Ultrastructural analysis of the hippocampus of the CTRL group (Figure 2a–c) showed normal tissue organization with well-preserved nerve and glial cells. Nerve extensions were well represented, rich in neurotubules and neurofilaments, with slight vacuolization at the axonal level. In addition, synapses were well represented and with well-preserved morphology.

Continuous aerobic training influenced hippocampal structure differently depending on the type of protocol performed by the animals.

**Figure 3:**
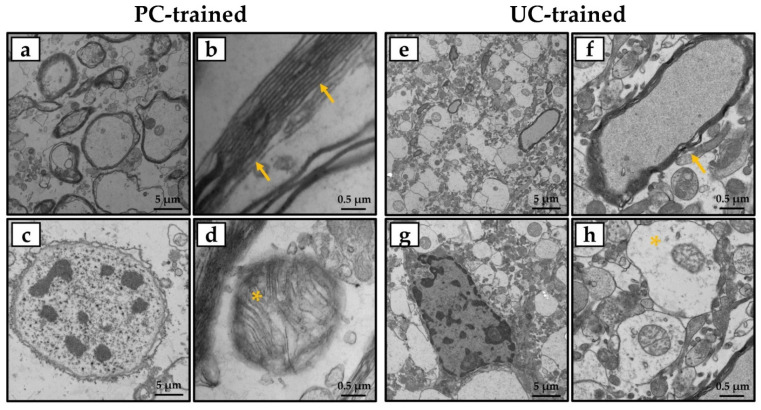
Transmission electron microscopy (TEM) evaluation of the hippocampus of trained mice. (**a**,**c**) Ultrastructural analysis of the hippocampus of PC-trained mice showed well-preserved tissue organization, with well-organized neuronal and glial cells and nerve processes rich in neurotubules and neurofilaments. (**b**,**d**) Numerous highly preserved myelin bundles (arrows) and mitochondria without morphological changes (asterisk) were observed. (**e**,**g**) Ultrastructural analysis of the hippocampus of UC-trained mice showed some morphological changes, such as slight vacuolization at the axonal level and few neurotubules and neurofilaments. (**f**,**h**) A reduced number of myelin bundles (arrow) and frequent mitochondrial alteration (asterisk) were detected. Scale bars represent 5 or 0.5 μm.

Particularly, ultrastructural analysis of the hippocampus of PC-trained mice (Figure 3a–d) showed features very similar to those of the CTRL group, with well-organized neuronal and glial cells and nerve processes rich in neurotubules and neurofilaments. In addition, brain tissue showed numerous highly preserved myelin bundles, and mitochondria were free of morphological changes. In contrast, some morphological changes were found in the brain tissue of UC-trained mice (Figure 3e–h), including a slight vacuolization caused by axonal swelling and a reduction in the number of neurotubules and neurofilaments. Finally, TEM evaluation showed a reduced number of myelin bundles and frequent mitochondrial alterations.

## 4. Discussion

Regular exercise induces profound health benefits for the body through mechanisms involving various physiological adaptations, including neural, immunological, vascular, and metabolic systems [35,36]. Interestingly, emerging data from studies in animal models and humans indicate that aerobic exercise benefits brain function and may prevent or delay the onset of neurodegenerative conditions by inducing structural and functional changes in the hippocampus, an area of the brain important for learning and memory [37,38]. Indeed, synaptic changes, which underlie cognitive processes, are known to depend on physiological mechanisms such as LTP, which is particularly present in the hippocampus [39]. Furthermore, it has been reported that the improvement in synaptic plasticity depends on the type of training provided [34,40]. Therefore, to better understand the mechanisms underlying the effects of aerobic exercise on the hippocampus and more generally on synaptic plasticity, in the present study we subjected young mice to two training protocols, PC and UC, differing in speed and speed variation.

First, we performed a functional evaluation by analyzing the effects of aerobic training on the synaptic plasticity expression by means of in vitro extracellular recordings in the CA1 region of mouse hippocampal slices. Our results showed that only the PC training protocol appeared to exert positive effects on synaptic plasticity throughout the electrophysiological recording, because we observed a significant increase in PS amplitude values after HFS compared to the other experimental groups. These data are in agreement with the results of our previous study, in which the administration of a PC training protocol has been shown to positively modulate hippocampal plasticity not only in young mice, but also reverses the blockage of the LTP induction phase typical of aged mice [41]. Additional scientific evidence confirms the beneficial effects of aerobic training on hippocampal synaptic plasticity. For example, Li et al. recently evaluated the effectiveness of a four-week aerobic training protocol on memory and the expression of proteins involved in synaptic plasticity in diabetic mice [42]. In addition to observing a significant reduction in fasting blood glucose and an improvement in insulin resistance, the authors found an increase in proteins associated with synaptic plasticity, pointing to aerobic exercise as a valid strategy to counteract the cognitive decline that characterizes diabetic mice [42].

In contrast, the UC training protocol did not induce any improvement in synaptic plasticity compared to sedentary mice of the same age. Particularly, PS amplitude values were significantly reduced in the first twenty minutes after HFS, whereas they reached values comparable to those of the CTRL group in the remaining time of electrophysiological recording. Notably, although the two trained groups did not differ significantly from the sedentary group after 65 min, a significant difference between them was found at the end of the electrophysiological recordings. This result suggests the importance of designing an appropriate training protocol to optimize the beneficial effects at the hippocampal level.

Electrophysiological data were confirmed by TEM evaluation, which showed that synaptic plasticity was affected differently depending on the type of protocol performed by the animals. Specifically, ultrastructural analysis of the hippocampus of PC-trained mice showed features very similar to those of the CTRL group, highlighting the presence of well-organized neuronal and glial cells and nerve processes rich in neurotubules and neurofilaments. Synapses were also well represented and with well-preserved morphology, in addition to the presence of numerous highly preserved myelin bundles and mitochondria without morphological changes. In contrast, the hippocampal tissue of the UC-trained mice exhibited some morphological changes, such as slight axonal vacuolization and a reduction in the number of neurotubules and neurofilaments, as well as a reduced number of myelin bundles and frequent mitochondrial changes.

In agreement with other experimental evidence, our results show that the benefits of exercise on cognitive function and neuroplasticity depend on the type of training protocol used. The underlying molecular and cellular mechanisms are not yet known. However, most scientific evidence agrees that the benefits of aerobic exercise may depend on an increase in growth factors and the increased expression of markers of synaptic plasticity, such as synaptophysin and postsynaptic density protein 95 (PSD-95) in the hippocampus [43]. In this context, the PI3K/AKT/mTOR pathway could play a crucial role, because exercise-induced activation of this pathway has been reported to promote the expression of PSD-95, improving memory performance [44,45]. Studies in rodents have also shown that early exercise increases axonal and neuronal density and improves the expression of BDNF and its tropomyosin-related receptor kinase B (TrkB) in hippocampal formation [11,46]. In agreement, Redila et al. observed that young, physically active rats show increased neurogenesis and dendritic arborization in the dentate gyrus compared to sedentary rats [47]. Interestingly, Serra and colleagues have suggested that exercise increases the expression of neurotrophic factors and stimulates neuronal growth, resulting in a neural reserve to be used in later life [48]. This hypothesis is supported by previous research in humans, which has shown a correlation between physical activity at an early age and long-term benefits on brain function [49].

## 5. Conclusions

Our data show that the use of an aerobic training protocol, such as the PC protocol, properly designed in terms of speed and speed variation, helps to maintain brain health and cognition. Interestingly, an adequate aerobic training protocol can induce important structural and functional changes in the hippocampus, the brain area responsible for learning and memory. This underlines the importance of physical exercise in counteracting age-related cognitive decline and suggests its key role in preventing the onset of cognitive impairment. Further studies will be required to understand the underlying cellular and molecular mechanisms, as well as the role of key biochemical mediators involved in the modulation of synaptic plasticity induced by aerobic training.

## Figures and Tables

**Table 1:** A schematic description of the two different aerobic exercise protocols used to train mice.

	PC Protocol	UC Protocol
Main features	Incremental speed changes with gradually increasing exercise intensity. Intensity increases in 2 RPM intervals from 10 to 32 RPM, with 12 speed changes	Single session training at 9 RPM, without speed changes
Training session duration	18 min	26 min
Weekly frequency	3 times a week	3 times a week
Training period	12 weeks	12 weeks

PC: progressive continuous; UC: uniform continuous; RPM: laps per minute.

## Data Availability

The data presented in this study are available on request from the corresponding author.
